# Athlete or Season? Gut Microbiota Variance in Elite Volleyball Players: A Compositional Performance-Based Reanalysis

**DOI:** 10.3390/sports14070292

**Published:** 2026-07-09

**Authors:** Junior Carlone, Giovanni Solarino, Saverio Giampaoli, Eugenio Alladio, Gioele Rosellini, Maurizio Cibba, Attilio Parisi, Alessio Fasano, Antonio Tessitore

**Affiliations:** 1 Department of Neurosciences, Biomedicine and Movement, University of Verona, 37134 Verona, Italy; 2 Department of Movement, Human and Health Sciences, University of Rome “Foro Italico”, 00135 Rome, Italy; 3 Division of Pediatric Gastroenterology and Nutrition, Mass General for Children and Harvard Medical School, Boston, MA 02114, USA; 4 Department of Chemistry, University of Turin, 10125 Turin, Italy; 5 Department of Nutrition, Harvard T.H. Chan School of Public Health, Boston, MA 02115, USA; 6 European Biomedical Research Institute of Salerno, 84121 Salerno, Italy; 7 Department of Economics, Engineering, Society, and Business Organization, University of Tuscia, 01100 Viterbo, Italy

**Keywords:** elite athletes, volleyball, gut microbiota, compositional data analysis, technical performance level

## Abstract

**Background:** The gut microbiota is an emerging factor in athletic performance. Methodological limitations persist in longitudinal microbiome studies, particularly regarding the compositional nature of microbiota data and the lack of standardized performance metrics in team sports. The present study applies a Compositional Data Analysis (CoDA) framework with ANOVA-Simultaneous Component Analysis (ASCA) to investigate longitudinal gut microbiota dynamics in elite volleyball athletes and introduces a novel Technical Performance Level (TPL) metric for objective performance quantification. **Methods:** Seven elite male volleyball athletes from the Italian SuperLega Championship were monitored across four sampling timepoints (T0, T1, T2, T3) during the Regular Season, Rest Period, and Tournament Period. Fecal samples underwent 16S rRNA metabarcoding. CLR-transformed data were analyzed at the Phylum, Family, and Genus level using ASCA with Season, Player, and their interaction. Statistical significance was assessed using the Freedman-Lane permutation test (10,000 iterations). Within-subject associations between TPL and CLR-transformed microbial features were assessed by repeated-measures correlation. **Results:** Season and Player effects were statistically significant at all taxonomic levels (*p* ≤ 0.0004), whereas the Season × Player interaction was significant at the Family and Genus levels (*p* ≤ 0.002) but not at the Phylum level (*p* = 0.108). The Player effect represented the largest source of variance at the Family (51.31%) and Genus (50.75%) levels. At the Phylum level, the Season × Player interaction accounted for the largest share of variance (36.23%), although it did not reach significance. Within-subject correlation analyses revealed no statistically significant association between microbial features and TPL at any taxonomic level, and none remained significant after correction for multiple comparisons. **Conclusions:** The application of a compositional data analysis framework modifies the interpretation of gut microbiota dynamics in elite volleyball athletes compared to standard approaches. Individual athlete identity accounted for most of the compositional variance in the microbiota, whereas exploratory within-subject analyses did not reveal statistically significant associations between taxa and TPL. Given the small cohort, any observed trends should be interpreted as exploratory and hypothesis-generating until confirmed in adequately powered cohorts. Longitudinal monitoring of gut microbiota could support individualized surveillance of gut health in elite sport.

## 1. Introduction

Elite athletic performance, among other factors, depends on the integration of metabolic, immunological, and neuromuscular systems, with the gut microbiota emerging as an unexpected but biologically compelling participant [1,2,3,4]. The microbiota-muscle axis has attracted growing scientific attention, with evidence linking microbial composition to energy substrate availability, inflammatory tone, and immune regulation in athletic populations [5,6,7,8,9,10]. These effects are mediated through multiple mechanisms, including short-chain fatty acids (SCFAs) production, B vitamin synthesis, and gut–brain axis signaling, all of which are physiologically relevant to elite sport [11,12,13,14,15,16,17]. Athletes generally exhibit greater microbial diversity and a higher abundance of SCFAs-producing taxa compared to sedentary individuals [18,19,20]. A preliminary study found that intestinal microbial communities maintain remarkable homeostatic stability across varying training and competition periods, despite significant fluctuations in the *Firmicutes/Bacteroidetes* ratio [21]. However, different training regimens and competition frequency can induce microbial shifts whose functional significance for health and performance outcomes remains poorly defined, particularly concerning technical performance efficiency [22,23]. In this regard, team sports like volleyball, which require explosive power, agility, and technical precision across variable competitive conditions, represent a particularly unexplored area of research [24].

Additionally, in microbiome research, despite the growing interest in recent years, a critical methodological gap persists. Most longitudinal studies in athletes have applied standard statistical approaches without accounting for the inherently compositional nature of microbiota data, potentially introducing bias and limiting interpretability [25,26]. Equally, objective and standardized metrics of technical athletic performance remain absent from microbiome research in team sports. Gut microbiota dynamics across competitive phases were previously characterized in this cohort, providing a preliminary basis for further investigation [21]. The present study introduces two methodological advances: a novel Technical Performance Level (TPL) metric derived from objective game statistics referenced to the entire Italian SuperLega championship population and a Compositional Data Analysis (CoDA) framework combined with ANOVA Simultaneous Component Analysis (ASCA), to investigate direct associations between microbiota composition and athletic performance in elite volleyball players.

The present study, therefore, aims to examine longitudinal associations between elite players’ gut microbiota composition and TPL using a methodologically CoDA-based approach, thereby contributing to the emerging field of precision sports medicine.

## 2. Materials and Methods

### 2.1. Study Design and Participants

This prospective longitudinal study followed 7 elite male volleyball athletes from the Italian SuperLega championship for 8 weeks during the 2023/2024 Competitive Season (Table 1). The international cohort included athletes born in France (*n* = 1), Italy (*n* = 4), and Serbia (*n* = 2). Inclusion criteria included age between 18 and 35 years, participation in the SuperLega championship, absence of antibiotic therapy in the preceding 6 months, and signed informed consent from players. Exclusion criteria included chronic gastrointestinal pathologies, use of probiotics, prebiotics, postbiotics, and restrictive diets during the preceding 6 months. The study protocol was approved by the Institutional Review Board of the University of Rome “Foro Italico” (CAR 240/2025). All participants provided written informed consent, and data collection adhered to both the principles of the Declaration of Helsinki and applicable national privacy regulations.

### 2.2. Data Collection Timeline

Data were collected across four timepoints during the In-Season competitive period: Regular Season (T0, T1), Rest Period (T2), and International Friendly Tournament (T3) (Table 1). Throughout the study, dietary adherence and gastrointestinal status remained stable, while some lifestyle factors varied across phases [21].

### 2.3. Technical Performance Level Assessment

**Figure 1:**
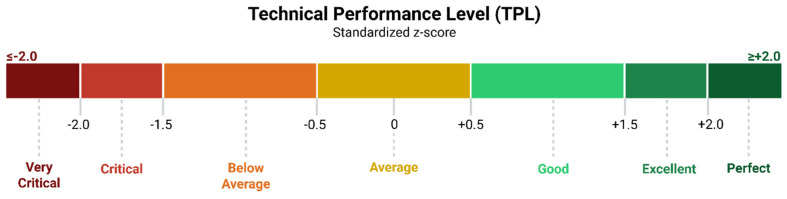
Z-score–based classification of Technical Performance Levels (TPL).

TPL was quantified using an integrated system of objective game metrics, developed based on established methodological models in the literature [27,28,29,30,31]. Original efficiency indices, specific to each athlete’s playing role, were employed: attack efficiency (% positive/negative attacks), serve efficiency (% positive/negative serves), and reception efficiency (% positive/negative receptions). For spikers and middle blockers (*n* = 6), attack, serve, and reception efficiencies were considered. For the Libero (*n* = 1), only reception efficiency was considered. For each available technical action, a z-score was computed as z = (athlete E% − SuperLega mean)/SuperLega SD, where athlete E% represents the athlete’s efficiency for that specific action in the individual match, while the mean and standard deviation of the 2023–2024 SuperLega (*n* = 176 athletes) are specific to role and action. Because the reference statistics are season-level averages, TPL absolute values and classification bands should be read as relative indicators within this cohort rather than as exact standard-deviation units. The composite TPL score was obtained as the mean of the z-scores of the available actions: TPL = (sum of z-scores)/n, where n is the number of actions effectively available for that athlete in that match. The three technical actions (attack, serve, reception) were weighted equally. For periods comprising multiple matches (Regular Season and International Friendly Tournament), TPL was calculated as the mean of the values obtained in the individual matches. For the Libero position, TPL corresponded directly to the reception efficiency value. TPL values were classified as follows: ≥+2.0, Perfect; +1.5 to <+2.0, Excellent; +0.5 to <+1.5, Good (above average); −0.5 to <+0.5, Average; −1.5 to <−0.5, Below Average; >−2.0 to <−1.5, Critical; ≤−2.0, Very Critical (Figure 1). Values exceeding the classification boundaries (>+2.0 or <−2.0) can occur, particularly in low-volume periods, because the efficiency percentage of a technical action is computed from a few observations (single attack, serve, or reception) and the resulting z-score is therefore sensitive to the number of actions performed (Table S1). The classification thresholds serve as interpretive anchors rather than hard boundaries.

### 2.4. Data Source and Microbiota Profiling

The microbiota sequencing data analyzed in the present study are derived from the same cohort and sampling timepoints reported in Carlone et al. [21]. Fecal samples were self-collected by participants with sterile swabs (COPAN, Brescia, Italy) immediately after a standardized training session, stored at −20 °C, and transported to the laboratory under temperature-controlled conditions at the four sampling timepoints (T0–T3; Table 1). The gut microbiota was profiled by 16S rRNA metabarcoding. DNA was extracted by mechanical lysis followed by purification [21]. To maximize reproducibility and minimize operator-dependent error, DNA extraction, library preparation, chip loading, and sequencing were performed on a fully automated robotic platform. Libraries were prepared on the Ion Chef Instrument (Thermo Fisher Scientific, Waltham, MA, USA) using Precision ID DL8 chemistry and the WG00607 16S Ion AmpliSeq™ custom primer pool, covering eight hypervariable regions (22 amplification cycles), barcoded with Precision ID IonCode adapters, and loaded onto Ion 540™ Chips. Sequencing was performed on the Ion GeneStudio™ S5 System (Thermo Fisher Scientific) using the “16S Metagenomics” application (Torrent Suite Software, 5.18.1). Taxonomic classification was performed with Ion Reporter Software (AmpliSeq Microbiome Health w1.3 workflow, version 5.20). Further wet-lab and bioinformatic details are reported in Carlone et al. [21].

### 2.5. Statistical Analysis

Descriptive statistics were performed in R (version 4.5.0), while CoDA, Principal Component Analysis (PCA), ASCA, and correlation analyses were performed in Python (version 3.13.3). Descriptive statistics are presented as mean ± standard deviation for continuous variables and frequencies for categorical variables. Microbiota data were analyzed exploiting the compositional data framework, CoDA. First, taxa were retained if present in at least 50% of subjects, where “detected in a subject” was defined as present in at least 50% of that subject’s timepoints. After prevalence filtering, compositional closure was restored. Prevalence filtering reduced data sparsity to 10.7% (Phylum), 12.7% (Family), and 9.2% (Genus). Residual zeros in the prevalence-filtered compositions were imputed by multiplicative replacement [32]. Each zero was replaced by 1/D^2^, where D is the number of retained taxa at a given level, and the non-zero parts were rescaled to preserve the closure. The centered log-ratio (CLR) transformation was then applied to project the simplex data into Euclidean space.

The global compositional structure was explored using PCA on CLR-transformed and Pareto-scaled data at the Phylum, Family, and Genus levels. To partition microbiota variance attributable to each experimental factor, ASCA was applied to CLR-transformed, Pareto-scaled data at the Phylum, Family, and Genus levels separately [33]. The model included Season, Player, and their interaction. The Season variable was defined at the phase level, with three levels: Regular Season (T0, T1), Rest Period (T2), and International Friendly Tournament (T3). These phases are defined by the competitive calendar and represent composite exposures encompassing training load, match density, recovery, and travel. They are therefore interpreted as integrated phase-level conditions rather than as an isolated biological Season effect. The player was explicitly included to account for repeated measures within individuals; ignoring it would have inflated the residual variance, masking the phase-associated variation.

Effect sub-matrices were computed using Type III sums of squares (SSQ). Statistical significance of each factor was assessed using the Freedman-Lane permutation procedure [34]. Briefly, for each factor, a reduced model excluding the term of interest was first fitted. Residuals from this reduced model were then permuted (10,000 iterations) and combined with the modeled factors to reconstruct permuted datasets under the null hypothesis. SSQ were recomputed at each iteration to build the empirical null distribution. Permutation *p*-values were computed using the plus-one (add-one) estimator, with 10,000 permutations; the minimum attainable value is therefore 1 × 10^−4^.

To address the repeated-measures structure for the within-subject terms, a player-restricted permutation sensitivity analysis was additionally performed, in which reduced model residuals were shuffled only within each player block. This restricted scheme provides the correct exchangeability for the within-subject terms (Season and Season × Player); however, it is not a valid null for the between-subject Player main effect, whose exchangeable unit is the whole subject and which is therefore tested only under the free scheme. The stability of ASCA loadings was assessed using bootstrap resampling (1000 iterations). Loadings were considered significant when their 95% confidence intervals did not include zero.

Within-subject associations between each CLR-transformed microbial feature and TPL were quantified by repeated-measures correlation, which estimates the common intra-individual association by fitting subject-specific intercepts with a single common slope [35]. One athlete accounted for all three missing TPL values and was excluded to permit within-subject estimation, yielding a cohort of 6 athletes and 24 observations. Because multiple taxa were tested at each taxonomic level (4 Phyla, 11 Families, and 14 Genera), *p*-values were adjusted by the Benjamini–Hochberg false discovery rate (FDR) procedure within each level. Given the cohort size, these correlations are regarded as exploratory; no sensitivity analyses of alternative zero-replacement strategies were performed, as such analyses would themselves be underpowered and could convey false precision.

## 3. Results

### 3.1. Sample Characteristics and Temporal Variations

**Figure 2:**
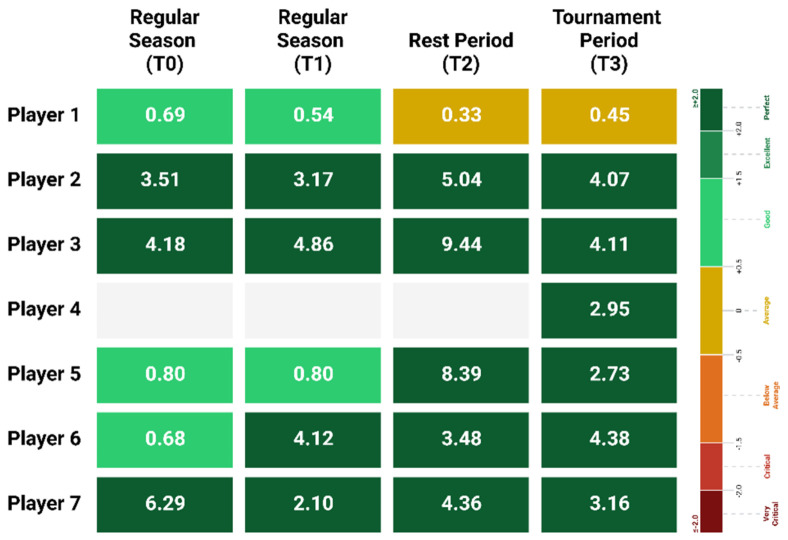
Heatmap of Technical Performance Level (TPL) z-scores for each player across competitive-load phases. Color intensity reflects TPL magnitude on a relative z-score scale. (Uncolored cells indicate missing data).

The final dataset comprised 28 observations from 7 athletes for microbiota profiling, collected across four sampling timepoints (T0, T1, T2, T3). For TPL, 25 observations were available, with 3 missing values, as one athlete did not participate in any match during the Regular Season or Rest Period (T0, T1, T2), despite training alongside the other athletes (Figure 2, Table 2).

### 3.2. Overall Compositional Structure

**Figure 3:**
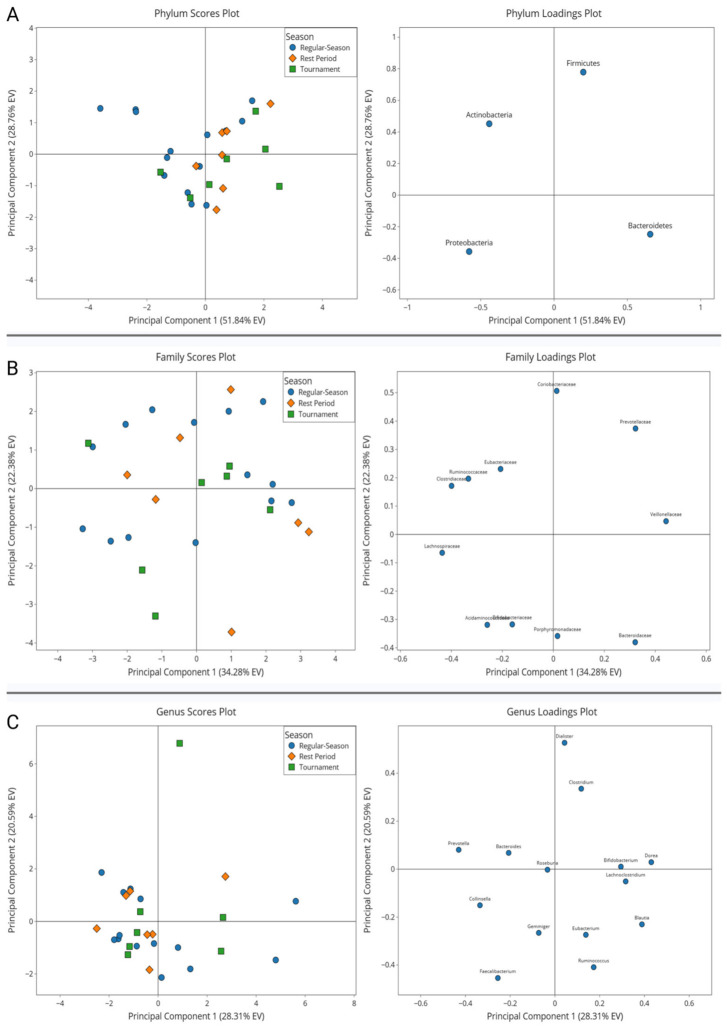
CoDA-PCA at Phylum (**A**), Family (**B**), and Genus (**C**) levels. For each taxonomic level, the Loadings Plot and Scores Plot are shown. Samples are color-coded by competitive phase. The extensive overlap across competitive phases in all Score Plots indicates that inter-individual variability outweighs phase-associated variation.

CoDA-PCA revealed a consistent compositional structure across all three taxonomic levels analyzed. At the Phylum level, PC1 (51.84% Explained Variance, EV) opposed *Firmicutes* and *Bacteroidetes* (+ = positive loadings) to *Actinobacteria* and *Proteobacteria* (− = negative loadings), while PC2 (28.76% EV) contrasted *Firmicutes* and *Actinobacteria* (+) with *Proteobacteria* and *Bacteroidetes* (−) (Figure 3A). At the Family level, PC1 (34.28% EV) opposed *Veillonellaceae, Prevotellaceae, Bacteroidaceae,* and *Porphyromonadaceae* (+) to *Lachnospiraceae, Clostridiaceae, Ruminococcaceae, Eubacteriaceae, Bifidobacteriaceae,* and *Acidaminococcaceae* (−), while PC2 (22.38% EV) was driven by *Coriobacteriaceae, Prevotellaceae, Eubacteriaceae, Ruminococcaceae*, and *Clostridiaceae* (+) against *Bacteroidaceae, Porphyromonadaceae, Bifidobacteriaceae,* and *Acidaminococcaceae* (−) (Figure 3B). At the Genus level, PC1 (28.31% EV) opposed *Dorea, Blautia, Lachnoclostridium, Bifidobacterium, Ruminococcus,* and *Eubacterium* (+) to *Prevotella, Bacteroides, Collinsella, Faecalibacterium,* and *Gemmiger* (−), while PC2 (20.59% EV) was driven upward by *Dialister* and *Clostridium* and downward by *Faecalibacterium, Ruminococcus, Eubacterium, Gemmiger,* and *Blautia* (Figure 3C). Across all three score plots, although samples from the Regular Season, Rest Period, and Tournament Period showed considerable overlap, Regular Season samples tended to distribute towards negative PC1 values, while Rest Period and Tournament Period samples shifted towards positive values, suggesting that inter-individual variability outweighed phase-associated variation, without excluding a phase-level effect on the overall compositional structure.

#### 3.2.1. Phylum Level

**Figure 4:**
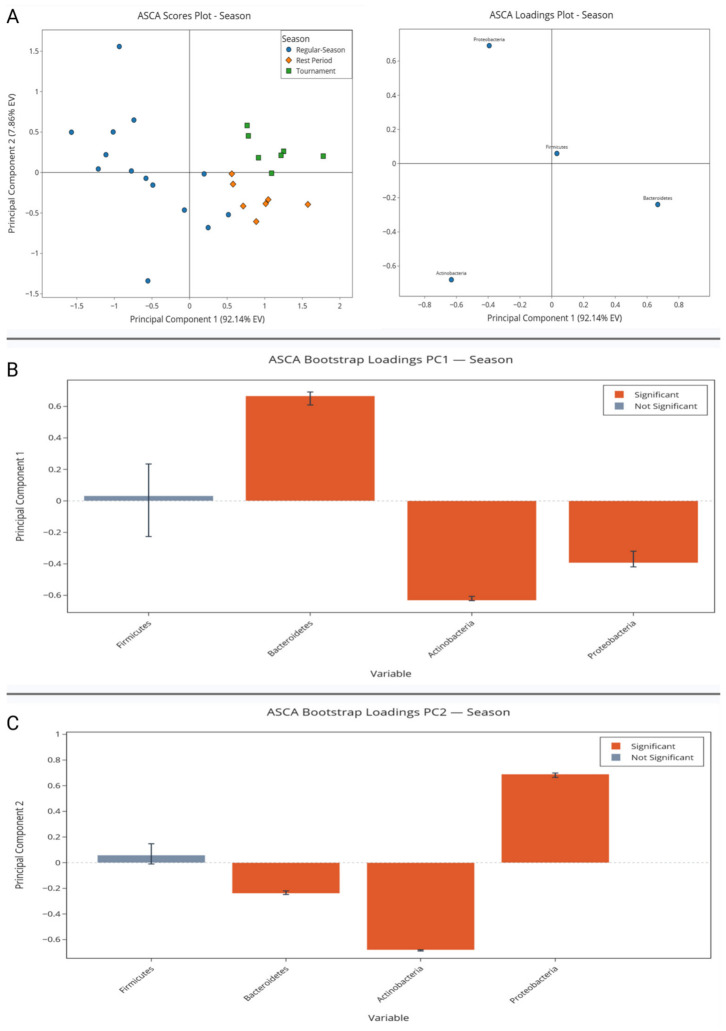
ASCA seasonal component at the Phylum level. (**A**) Score Plot and Loadings Plot of the Season component: PC1 (92.14% EV) separates the Regular Season (−) from the Rest Period and Tournament (+); PC2 (7.86% EV). *Bacteroidetes* is the strongest positive driver; *Actinobacteria* and *Proteobacteria* are the strongest negative drivers. (**B**) Bootstrap Loadings PC1: *Bacteroidetes* significantly positive; *Actinobacteria* and *Proteobacteria* significantly negative; *Firmicutes* non-significant. (**C**) Bootstrap Loadings PC2: *Proteobacteria* significantly positive; *Bacteroidetes* and *Actinobacteria* significantly negative; *Firmicutes* non-significant.

ASCA of the seasonal component at the Phylum level revealed that PC1 explained 92.14% of the variance attributable to season, with Regular Season samples clustering on the negative side of PC1 and Rest Period and Tournament Period samples shifting towards positive values (Figure 4A). Bootstrap-validated loadings on PC1 identified *Bacteroidetes* as the strongest significant positive driver (+), indicating its relative enrichment during the Rest Period and Tournament phases, while *Actinobacteria* (−) and *Proteobacteria* (−) displayed significant negative loadings, suggesting their relative enrichment during the Regular Season. *Firmicutes* did not reach significance on PC1 (Figure 4B). On PC2 (7.86% EV), *Proteobacteria* represented the strongest significant positive driver (+), while *Actinobacteria* (−) and *Bacteroidetes* (−) showed significant negative loadings. *Firmicutes* was again non-significant (Figure 4C).

No Phylum-level within-subject association with TPL was significant under repeated-measures correlation, and none survived FDR correction. *Bacteroidetes* (r = 0.400, *p* = 0.089, q = 0.186) and *Proteobacteria* (r = −0.396, *p* = 0.093, q = 0.186) showed the strongest positive and negative coefficients. *Actinobacteria* (r = −0.153, *p* = 0.531) and *Firmicutes* (r = −0.023, *p* = 0.926) showed no covariation with TPL.

#### 3.2.2. Family Level

**Figure 5:**
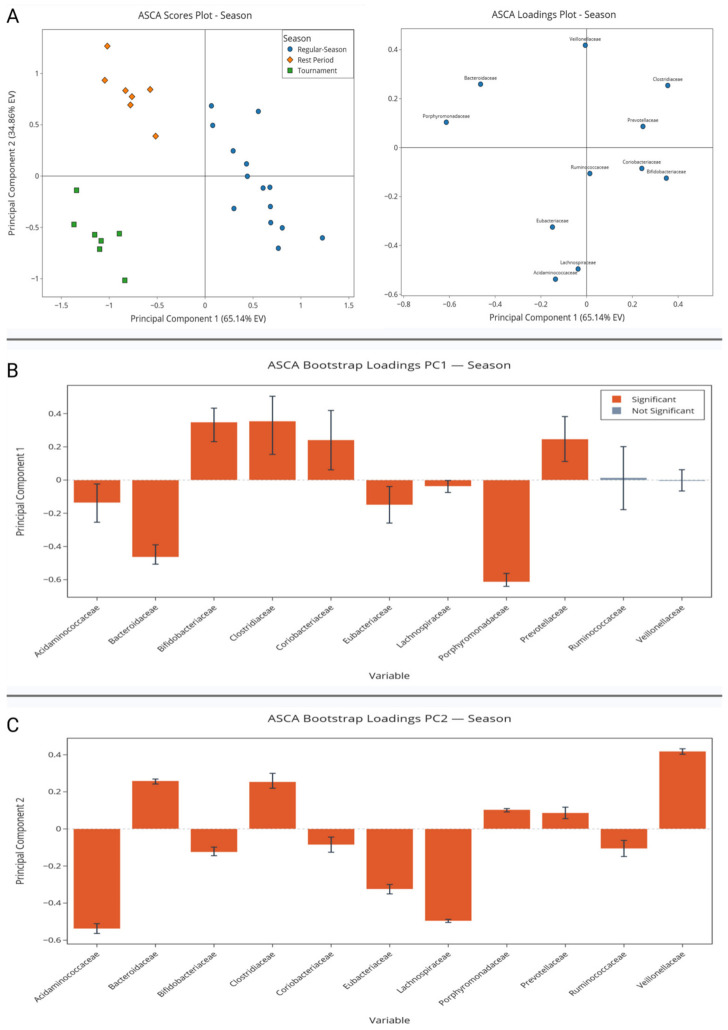
ASCA seasonal component at the Family level. (**A**) Score Plot and Loadings Plot of the Season component: PC1 (65.14% EV) separates the Rest Period (−) from the Regular Season (+); PC2 (34.86% EV) further distinguishes timepoints along the second component. *Porphyromonadaceae* and *Bacteroidaceae* are the strongest negative drivers; *Bifidobacteriaceae* and *Clostridiaceae* are the strongest positive drivers. (**B**) Bootstrap Loadings PC1: *Porphyromonadaceae*, *Bacteroidaceae*, and *Eubacteriaceae* significantly negative; *Bifidobacteriaceae*, *Clostridiaceae*, *Prevotellaceae*, and *Coriobacteriaceae* significantly positive. (**C**) Bootstrap Loadings PC2: *Veillonellaceae*, *Bacteroidaceae*, and *Clostridiaceae* are significantly positive; *Acidaminococcaceae* and *Lachnospiraceae* are significantly negative.

ASCA of the seasonal component at the Family level revealed that PC1 explained 65.14% of the variance attributable to season, with Rest Period samples clustering in the negative region of PC1 and Regular Season samples in the positive region (Figure 5A). Bootstrap-validated loadings on PC1 identified *Porphyromonadaceae* as the strongest significant negative driver (−), followed by *Bacteroidaceae* (−), *Eubacteriaceae* (−), and *Acidaminococcaceae* (−), indicating their relative enrichment during the Rest Period and Tournament Period. *Bifidobacteriaceae* (+), *Clostridiaceae* (+), *Prevotellaceae* (+), and *Coriobacteriaceae* (+) displayed significant positive loadings, indicating relative enrichment during the Regular Season. *Lachnospiraceae* showed a small but significant negative loading on PC1 (−), while *Ruminococcaceae* and *Veillonellaceae* did not reach significance on this component (Figure 5B). On PC2 (34.86% EV), all families displayed significant loadings. *Veillonellaceae* represented the strongest positive driver (+), followed by *Bacteroidaceae* (+), *Clostridiaceae* (+), *Porphyromonadaceae* (+), and *Prevotellaceae* (+). On the negative side, *Acidaminococcaceae* (−) and *Lachnospiraceae* (−) showed the most pronounced loadings, followed by *Eubacteriaceae* (-), *Bifidobacteriaceae* (−), *Ruminococcaceae* (−), and *Coriobacteriaceae* (−) (Figure 5C).

No Family-level association with TPL was significant under repeated-measures correlation, and none survived FDR correction. *Lachnospiraceae* showed the largest coefficient (r = −0.193, *p* = 0.427, q = 0.955), followed by *Bacteroidaceae* (r = 0.151, *p* = 0.538) and *Acidaminococcaceae* (r = −0.133, *p* = 0.588).

#### 3.2.3. Genus Level

**Figure 6:**
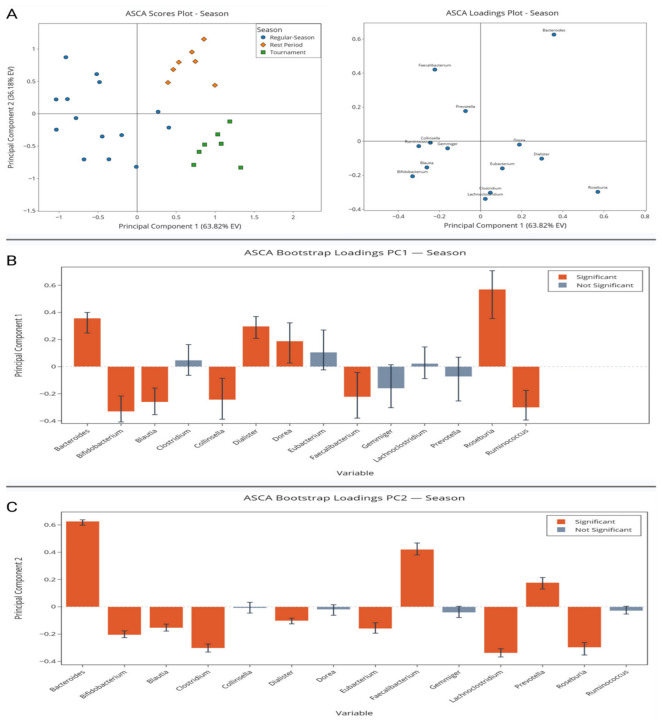
ASCA seasonal component at the Genus level. (**A**) Score Plot and Loadings Plot of the Season component: PC1 (63.82% EV) separates the Regular Season (−) from the Rest Period and Tournament (+); PC2 (36.18% EV) further distinguishes the Rest Period (+) from the Tournament (−). *Roseburia, Bacteroides*, and *Dialister* are the strongest positive drivers on PC1; *Ruminococcus, Bifidobacterium*, and *Blautia* are the strongest negative drivers. (**B**) Bootstrap Loadings PC1: *Roseburia, Bacteroides,* and *Dialister* are significantly positive; *Ruminococcus, Bifidobacterium, Blautia, Faecalibacterium,* and *Collinsella* are significantly negative. (**C**) Bootstrap Loadings PC2: *Bacteroides* and *Faecalibacterium* are significantly positive; *Lachnoclostridium*, *Clostridium*, and *Roseburia* are significantly negative.

The ASCA Score Plot at the Genus level for the Season component revealed a separation of Regular Season samples (−PC1) from Rest Period and Tournament samples (+PC1), with the Rest Period and Tournament Period further distinguished along PC2 (+ and −, respectively). PC1 explained 63.82%, and PC2 explained 36.18% of the variance attributable to Season (Figure 6A). Bootstrap-validated loadings on PC1 identified as significant during the Rest Period and Tournament Period: *Roseburia* (+), *Bacteroides* (+), *Dialister* (+), and *Dorea* (+); whereas during the Regular Season, *Ruminococcus* (−), *Bifidobacterium* (−), *Blautia* (−), *Faecalibacterium* (−), and *Collinsella* (−) were significantly enriched. *Clostridium, Gemmiger, Lachnoclostridium, Eubacterium*, and *Prevotella* did not reach significance on PC1 (Figure 6B). On PC2, *Bacteroides* (+), *Faecalibacterium* (+), and *Prevotella* (+) showed significant positive loadings, while *Lachnoclostridium* (−), *Clostridium* (−), *Roseburia* (−), *Bifidobacterium* (−), *Blautia* (−), *Eubacterium* (−), and *Dialister* (−) showed significant negative loadings (Figure 6C). *Collinsella, Dorea, Gemmiger,* and *Ruminococcus* were non-significant on PC2 (Figure 6C).

No Genus-level association with TPL was significant under repeated-measures correlation, and none survived FDR correction. *Bacteroides* showed the strongest positive coefficient (r = 0.349, *p* = 0.143, q = 0.900) and *Lachnoclostridium* the strongest negative (r = −0.337, *p* = 0.158, q = 0.900), followed by *Roseburia* (r = 0.268, *p* = 0.267).

#### 3.2.4. Cross-Taxonomic Variance Partitioning

ASCA variance partitioning results at the Phylum, Family, and Genus levels are summarized in Table 3. Due to the unbalanced design and Type III SSQ, terms are non-additive, and explained variance percentages may not sum to 100%. Across all three taxonomic levels, Season and Player terms were statistically significant (*p* ≤ 0.0004). The Season × Player interaction was significant at the Family and Genus levels but not at the Phylum level (*p* = 0.108). At the Family and Genus levels, Player emerged as the largest source of variance (51.31% and 50.75%), followed by the Season × Player interaction (27.97% and 31.62%) and Season (6.86% and 4.30%). The Season effect, although statistically significant across all taxonomic levels, explained a numerically smaller proportion of variance at the Family and Genus levels compared to the Phylum level.

## 4. Discussion

Building on Carlone et al., the present study extends the variance partitioning analysis by applying it within a compositional data framework [21]. Given the small cohort size, the findings should be interpreted as exploratory and hypothesis-generating. By applying a CLR transformation prior to ASCA, the analysis accounts for the constrained nature of relative abundance data, in which taxa are not independent, and their values sum to one. This approach allows variance decomposition to operate in Euclidean space, providing a more resolved characterization of the sources of microbiota variability across competitive phases. The practical consequence is that Season and Player ASCA terms at each taxon level were statistically significant (*p* < 0.001). Player accounted for most of the decomposition at the Family and Genus levels, consistent with the overall microbial community stability reported by Carlone et al. [21]. Although the seasonal component explained a modest to moderate proportion of total variance across taxonomic levels (Phylum 14.86%, Family 6.86%, Genus 4.30%), the ASCA Score Plot, particularly at the Family and Genus levels, displayed a separation of the three competitive phases along PC1 of the Season effect matrix. This separation is an intrinsic and expected property of ASCA. PCA of the CLR data shows that, at every level, the total variance is outweighed by inter-individual and residual variation, so the phase-associated variation is buried. ASCA instead decomposes the data variance and displays the Season effect matrix alone, from which Player and residual variation have been removed. This pattern suggests that, although quantitatively minor relative to individual identity, phase-associated variation produces systematic, biologically interpretable phase-specific compositional modulations, consistent with the dynamics described by Carlone et al. [21]. The present study, therefore, represents a methodological advancement, oriented towards the future directions identified by Carlone et al., confirming its fundamental biological patterns and refining the sources of variance through a compositional framework [21]. The adoption of a compositional data analytical approach further refined the observed microbial dynamics, highlighting the pivotal role of an appropriate statistical approach in longitudinal microbiome research, a rapidly expanding field within the Sport Microbiome area.

The application of prevalence filtering and CLR transformation in the present reanalysis provides a more conservative and compositionally appropriate evaluation of taxon-level dynamics. *Rikenellaceae*, which showed significant enrichment during the Rest Period in Carlone et al. using one-way ANOVA on autoscaled relative abundances, did not reach significance within the CoDA-ASCA framework, reflecting the more conservative prevalence filtering and permutation-based inference applied here rather than an absence of biological signal [21]. This observation underscores how the choice of analytical pipeline can substantially influence taxon-level findings, and highlights *Rikenellaceae* as a candidate taxon warranting targeted investigation in larger, adequately powered cohorts.

Beyond the variance partitioning structure, within-subject correlation analyses were conducted to examine whether temporal fluctuations in gut microbiota composition covaried with TPL within individual athletes. No association reached statistical significance at any taxonomic level, and none survived Benjamini–Hochberg FDR correction. At the Phylum level, *Proteobacteria* showed the strongest negative coefficient, and at the Genus level, *Lachnoclostridium* showed the strongest negative coefficient; neither reached significance. Both taxa have previously been discussed in relation to intestinal ecology and host physiological states. However, these descriptive trends should not be interpreted as evidence of an association between these microbial features and TPL, particularly given the small cohort size [21,36,37].

Taken together, the within-subject correlation analysis indicates that, although individual athlete identity accounts for the overall compositional variance, no microbial taxon covaried significantly with TPL within individuals in this cohort. These findings support a predominantly non-associative interpretation of the relationship between gut microbiota composition and technical performance at the individual level, and any observed trends should be regarded as exploratory only and warrant confirmation in adequately powered longitudinal studies before any biological or practical interpretation is attempted.

### 4.1. Practical Application

Although the present study did not identify significant within-subject associations between gut microbiota composition and TPL, the predominance of inter-individual variance identified by ASCA indicates that longitudinal intra-athlete monitoring, rather than cross-sectional between-group comparisons, remains the most appropriate paradigm for identifying clinically meaningful microbiota signals in elite sport. Longitudinal microbiota profiling could complement existing monitoring tools as part of a broader, individualized approach to athlete health surveillance, although this would require integration with markers not assessed in the present study, such as gut permeability, inflammatory biomarkers, gastrointestinal symptoms, and recovery indices [38,39,40,41]. For instance, dietary strategies targeting key microbial taxa, such as increasing dietary fiber and fermented food intake to support SCFAs-producing communities, may help maintain overall gut health during periods of highly competitive demand, even in the absence of demonstrated direct effects on performance [17,42,43,44,45]. However, the lack of a consensus definition of an optimal gut microbiota profile currently limits the interpretation of microbiota-derived biomarkers [46,47]. Incorporating gut microbiota monitoring throughout the competitive season could represent a possible strategy for high-level teams [48,49,50]. Furthermore, given the widespread availability of match analysis in elite volleyball, the longitudinal integration of TPL data from each athlete’s previous seasons could represent an innovative approach to individual performance-monitoring protocols.

### 4.2. Strengths and Limitations

The present study offers several methodological strengths. The application of a CoDA framework combined with ASCA represents a methodological advancement over the standard approaches previously applied to this dataset, enabling partitioning of microbiota variance attributable to season, individual athlete identity, and their interaction, while accounting for the compositional nature of microbiota data. Freedman-Lane permutation testing was used to assess statistical significance, avoiding reliance on distributional assumptions that might be difficult to justify with small sample sizes. Bootstrap validation of ASCA loadings provided robust statistical inference at the univariate level of interpretation. The introduction of the TPL metric constitutes an original methodological contribution, offering an objective, role-specific quantification of technical performance based on match statistics referenced to the entire SuperLega Championship population. The within-subject longitudinal design further strengthens the study by reducing inter-individual variability and improving the interpretability of temporal microbiota dynamics. Finally, the multi-taxonomic approach, with simultaneous analysis at the Phylum, Family, and Genus levels, provides a comprehensive compositional characterization of variations across the competitive season.

However, several limitations must be acknowledged. The small sample size limits statistical power to detect modest associations between microbiota composition and TPL. As an exploratory first investigation, no a priori power calculation was performed. Consequently, the findings regarding microbiota-TPL associations should be considered preliminary and warrant confirmation in larger, adequately powered studies. The strongest trend, a non-significant negative within-subject association between *Proteobacteria* and TPL, requires confirmation in adequately powered cohorts before any mechanistic interpretation is warranted. Furthermore, the within-subject correlation analysis was conducted on a reduced cohort of six athletes, as one athlete with TPL data available for a single timepoint was excluded to enable within-subject centering [51]. The correlational design does not allow causal relationships between microbiota and performance to be established. A further limitation concerns the TPL metric, which should be considered an exploratory composite index rather than a formally validated measure of volleyball performance. Its calculation does not account for action volume, and no minimum-volume threshold was applied. Values derived from a few technical actions may therefore be unstable, and the metric may also be influenced by role-specific characteristics. This warrants cautious interpretation and further validation, including volume-adjusted scoring, in larger cohorts. Absolute TPL values are intended only as relative indicators; the within-subject analysis is unaffected, as repeated-measures correlation centers each athlete.

The player was modeled as a factor with seven specific athletes, and therefore, the results are tied to this cohort. Consequently, ASCA decomposition is close to saturation given the limited degrees of freedom. In this setting, the partitioning of variance remains informative for identifying main sources of variability, but the exact percentages should not be over-interpreted. Because the competitive phases are calendar-defined composites, partly sampled at a single timepoint, the associated shifts cannot be attributed to a single factor, and phase-level inference remains exploratory. The exclusive use of 16S rRNA metabarcoding precludes direct functional characterization of microbial metabolic pathways [49]. However, this approach remains suited to longitudinal monitoring designs, where its lower cost, standardized protocols, and adequate sensitivity for tracking taxonomic composition at the genus level represent relevant methodological advantages [50]. The absence of inflammatory biomarkers and measures of intestinal permeability prevents direct links between the observed microbial changes and physiological outcomes [52,53]. Furthermore, the study covers a portion of a single competitive season in a single team sport among elite male athletes, limiting generalizability to other sports, sexes, and competitive contexts.

### 4.3. Future Directions

Validation studies in larger cohorts and across complete competitive seasons are needed to confirm the robustness of the Player effect and the identified compositional seasonal modulations. The generalizability of the TPL metric should be evaluated across different volleyball roles, competitive contexts, other team sports, and broader elite populations, while future studies should further assess its robustness through volume-adjusted and alternative scoring approaches. The integration of shotgun metagenomics sequencing will enable functional characterization of the microbiota across different competitive phases [49,50]. Finally, the leading Player effect identified by the CoDA-ASCA framework suggests that predictive models based on individual microbiota profiles represent a promising avenue for personalized athlete monitoring, although their practical utility requires validation in larger longitudinal cohorts and integration with physiological and performance-related outcomes.

## 5. Conclusions

This study demonstrates that applying a compositional data analysis framework comprising the CLR transformation and ASCA alters the interpretation of gut microbiota dynamics in elite volleyball athletes compared with standard relative abundance-based approaches. The present study introduces TPL as an original, role-specific metric for the objective quantification of athletic performance in team sports, derived from real match statistics referenced to the entire SuperLega championship. Individual athlete identity was the largest source of microbiota variance at the Family and Genus levels, whereas at the Phylum level, the largest share of variance was attributable to the Season × Player interaction, which, however, did not reach significance. In all models, the Season main effect alone accounted for the smallest portion of variance. Exploratory within-subject correlation analyses did not reveal statistically significant associations between TPL and microbial taxa. These observations highlight that the choice of statistical framework determines the conclusions that can be drawn from longitudinal microbiota data, and that individual microbial identity appears to outweigh seasonal or performance-related signals in this population, although seasonal periods can produce a systematic effect on the gut microbiota.

We propose that longitudinal gut microbiota monitoring be considered a methodological approach for future research on individualized athlete health in elite volleyball, rather than a validated tool for recovery optimization or performance prediction. Future studies with larger samples, compositional analytical frameworks, validated TPL metrics, and controlled interventional designs are needed to determine whether targeted microbiota modulation can translate into measurable performance benefits in elite volleyball.

## Figures and Tables

**Table 1:** Study design and sampling program. The characteristics shown for T0–T3 refer to the specific monitoring period associated with each sampling timepoint.

Period	Phase	Duration	Training Hours	Matches	Date	Tournament
T0	Regular Season	1 Week	20	1	5 February 2024	SuperLega Championship
T1	Regular Season	3 Weeks	55	4	26 February 2024	SuperLega Championship
T2	Rest Period	2 Weeks	15	1	14 March 2024	SuperLega Championship
T3	International Friendly Tournament	10 Days	10	5	4 April 2024	International Friendly Tournament

**Table 2:** Athletes’ technical performance level across competitive phases.

Variables	Value (Mean ± SD)	Value (Mean ± SD)	Value (Mean ± SD)	Value (Mean ± SD)
Time	Regular Season (T0)	Regular Season (T1)	Rest Period (T2)	International Friendly Tournament (T3)
TPL	2.69 ± 2.34	2.85 ± 2.71	5.22 ± 3.32	3.12 ± 1.34

(TPL = Technical Performance Level).

**Table 3:** ASCA variance partitioning at Phylum, Family, and Genus levels. Statistical significance assessed by Freedman-Lane permutation testing (10,000 permutations); *p*-values for Season and Season × Player are from the player-restricted permutation, and those for Player from the free permutation.

Term	Phylum: Sum of Squares	Phylum: Explained Variance	Phylum: *p*-Value	Family: Sum of Squares	Family: Explained Variance	Family: *p*-Value	Genus: Sum of Squares	Genus: Explained Variance	Genus: *p*-Value
Season	16.05	14.86%	0.0004	20.37	6.86%	0.0001	16.23	4.30%	0.0001
Player	33.04	30.59%	0.0001	152.38	51.31%	0.0001	191.83	50.75%	0.0001
Season × Player	39.13	36.23%	0.108	83.09	27.97%	0.002	119.52	31.62%	0.0003
Residual	21.26	19.68%	Not Applicable	26.16	8.81%	Not Applicable	33.88	8.96%	Not Applicable

## Data Availability

Athlete performance data are not publicly available for privacy reasons, but are available from the corresponding author upon reasonable request and prior Institutional Review Board approval. The source code is available from the corresponding author upon reasonable request.

## Supplementary Materials

The following supporting information can be downloaded at: https://www.mdpi.com/article/10.3390/sports14070292/s1, Table S1. Skill action counts by Player and Time Point.
