# Supplementary material for: Athlete or Season? Gut Microbiota Variance in Elite Volleyball Players: A Compositional Performance-Based Reanalysis
Source: Sports (Basel). 2026 Jul 9;14(7):292. doi: 10.3390/sports14070292 (PMC13416989; doi:10.3390/sports14070292)
Supplement: Supplementary file 1 [file sports-14-00292-s001.zip › sports-4397807-supplementary.pdf]

Player	Role	Period	Attack (n)	Serve (n)	Reception (n)
1	Libero	T0	N/A	N/A	16
1	Libero	T1	N/A	N/A	57
1	Libero	T2	N/A	N/A	12
1	Libero	T3	N/A	N/A	95
2	Spiker	T0	31	17	3
2	Spiker	T1	112	53	14
2	Spiker	T2	21	14	4
2	Spiker	T3	115	55	10
3	Spiker	T0	9	10	17
3	Spiker	T1	71	46	95
3	Spiker	T2	4	3	2
3	Spiker	T3	45	24	34
4	Spiker	T0	0	0	0
4	Spiker	T1	0	0	0
4	Spiker	T2	0	0	0
4	Spiker	T3	9	1	10
5	Middle Blocker	T0	0	17	1
5	Middle Blocker	T1	5	1	2
5	Middle Blocker	T2	7	3	1
5	Middle Blocker	T3	6	15	0
6	Middle Blocker	T0	6	11	0
6	Middle Blocker	T1	30	54	7
6	Middle Blocker	T2	9	9	1
6	Middle Blocker	T3	40	64	3
7	Middle Blocker	T0	7	17	2
7	Middle Blocker	T1	24	42	10
7	Middle Blocker	T2	5	4	1
7	Middle Blocker	T3	38	50	9

(N/A = Not Applicable)

Table S1. Skill action counts by Player and Time Point.
